# Healthcare associated pathogens in a changing world

**DOI:** 10.1186/1824-7288-40-S1-A5

**Published:** 2014-08-11

**Authors:** Caterina Mammina, Daniela Maria Geraci, Laura Saporito, Giorgio Graziano, Marco Scognamillo, Celestino Bonura, Mario Giuffrè

**Affiliations:** 1 Department of Sciences for Health Promotion and Mother-Child Care “G. D’Alessandro”, University of Palermo, Palermo, Italy

## 

In developed countries about 10% of the hospitalizations are complicated by a healthcare-associated infection [1]. Up to 75% of these infections are due to multidrug-resistant organisms (MDROs) [1]. Antimicrobial resistant bacterial infections are associated to higher morbidity, mortality and healthcare costs than those caused by susceptible organisms [1]. The findings of the point prevalence survey in European acute care hospitals published in 2013 by the European Centre for Disease Control and Prevention (ECDC) show large variations between countries and between different regions of the same country, with Italy being allocated within the high-endemic areas for both MRSA and MDROs [2].

Despite antimicrobial resistance affects most bacterial species, MDR Gram negatives represent the most serious threat. In a few years *Enterobacteriaceae*, mainly *Escherichia coli* and *Klebsiella pneumoniae*, have evolved from extended spectrum β-lactamase (ESBL) producing to carbapanem-resistant organisms [3]. Simultaneously, *Acinetobacter baumannii* has quickly become extremely or pan-drug resistant [4]. Carbapenem resistant Gram negatives heavily impact on clinical outcomes with mortality rates significantly higher than the susceptible strains of the same species [1]. Of further concern, very few antimicrobial agents are available for an effective treatment of these infections and new agents active against these organisms are not currently in development.

Many intertwining factors are driving these epidemiological changes, involving patients, healthcare delivery systems, infection control practices and, most important, misuse and inappropriate use of antibiotics in all healthcare facilities, in community and in animal husbandry. In particular, the transition of the healthcare delivery systems from a hospital-centered model to a healthcare facility network has gradually blurred the borders between hospital and community and the patients’ travel within this network has critically contributed to disseminate MDROs [5]. As a consequence, antimicrobial resistance is now as common, if not more so, in post-acute clinical facilities, such as long term care settings and nursing homes [5]. The “revolving door” is the very efficacious image used as the paradygm of the spreading routes of organisms with hospital and community reservoirs, as *E. coli* or MRSA. The revolving door, indeed, enlightens how the colonized patients entering back and forth several healthcare settings drive the amplification of the antibiotic resistance [6].

Stringent infection control and prevention practices and wise use of antibiotics are unanimously agreed as the key actions to fight MDROs. Of course, we need new antibiotics, but first we have to learn how to protect them from a precipitous erosion of their effectiveness.
